# Cases of the Spontaneous Cure of Aneurism, with Remarks

**Published:** 1788

**Authors:** Edward Ford

**Affiliations:** Surgeon to the Westminster General Dispensary


IV. *Cases of the spontaneous Cure of Aneurism, with Remarks. Communicated in a Letter to Dr.* Simmons, *by Mr.* Edward Ford, *Surgeon to the Westminster General Dispensary.*

AN aneurism of the larger vessels, when it occurs in the trunk of the body, is a disease that is usually fatal; and it is not uncommonly so when it happens in the extremities; the mode of cure in the latter case, whether by amputation of the limb, or by tying the artery, being universally allowed to be hazardous. These considerations have induced me to request of you a place in the London Medical Journal for the following cases and observations, written with a view to draw the attention of the public to the efforts nature sometimes spontaneously makes towards relieving herself of this disease. These efforts, although they have not altogether escaped the notice of medical practitioners, have, in this country, not been much attended to. Future investigation will probably throw such a light on this important branch of the pathology of aneurism as may lead to great practical improvements. The cases I now communicate to you, serve to establish the fact, that in cases of aneurism, rifm, the efforts of nature alone, unaffifted by art, have produced, in the coats of the veffel, a coalefcence of its fides firm enough to render the artery impervious to the impetus of the blood, whilft the circulation in the extremity has been amply fupported by the collateral branches going off above the aneurifmal tumour.

The firft opportunity of becoming acquainted with this fact, occurred to me, feveral years ago, in the cafe of a chairman who applied to me at the Weftminfter Difpenfary for a fwelling in the ham, which was evidently a popliteal aneurifm, and was confidered as fuch by the Phyficians of the Difpenfary as well as by feveral furgeons of eminence. The patient was afterwards admitted into an hofpital; and, at the end of three months, when he called upon me, I found that the tumour had totally difappeared, and that the limb was wafted, and a little weaker than the other, but that he was capable of doing his bufinefs. Upon inquiry I could not learn that the cure could be afcribed to any other means, than to the efforts of nature, with which an horizontal pofition of the body and a regular diet might perhaps have cooperated.

This

This man died foon after, of a fever; and as the limb was not examined by diffection, and a doubt arofe whether the tumor was aneurifmal, or not, the circumftances of the cafe were not deemed ftrong enough to juftify any inference to be made from it; but they ferved to ftimulate me to a clofe attention to the difeafe, and in the following cafe I had an opportunity of gaining a further infight into this remarkable phenomenon.

### CASE I.

JOHN CATHAY, thirty-fix years of age, of great St. Andrew's ftreet, Seven Dials, applied to me for the cure of a tumour fituated on the anterior and upper part of the right thigh, about three inches below Poupart's ligament. It was of the fize of a turkey's egg and had a ftrong pulfation. He faid that it had been increafing feveral weeks. The limb, at this time, had undergone no farther alteration, and he walked apparently with eafe. He fhewed me, at the fame time, a fwelling of about the fize of a pullet's egg in the ham of the other leg, in which was felt a tremulous pulfation; but this, he faid, was attended with no inconvenience. He was a healthy-looking man, and followed the bufinefs of a cloaths-preffer, an employment that requires a ftrong exertion of the mufcles both of the upper and lower extremities.

I had fo little to fay to him, which could adminifter the fmalleft degree of comfort, that I faw no more of him for two months, when I was requefted to vifit him, and found him in bed, with a burning fkin, a quick, full pulfe, difficult refpiration, and a tendency to delirium. The fwelling of the right thigh, the dimenfions of which were now very much increafed, was covered with a cataplafm, compofed chiefly of muftard feed, which had been applied by an empiric, with a view to promote the fuppuration; and a cordial regimen had been directed for the fame purpofe.

The antiphlogiftic plan, which feemed to be fo obvioufly indicated, was now adopted, and with fo good effect, that in a few days the fever was abated, and the local inflammation, occafioned by the irritating application, in fome degree palliated; ftill, however, there remained an immenfe tumor extending from the groin downwards almoft to the knee, ftrongly pul-

 fating,

ſating, and ſomewhat inflamed on its ſurface.

The patient was now ſeen by Mr. Andree, Mr. Cruickſhank, Mr. Adair Hawkins, Mr. John Howard, Mr. Vaux, and the late Mr. Juſtamond, all of whom agreed with me in opinion that no operation of ſurgery could, at this period of the diſeaſe, give him a probable chance of recovery. We now examined the other leg, but found no traces left of the ſwelling I had formerly ſeen. This tumor, which I ſhall now venture to call a popliteal aneuriſm (as the ſubſequent diſſection proved it to be ſuch) had totally diſappeared; but the knowledge of its having exiſted, and the conviction ſuch knowledge carried with it that a diſpoſition to this diſeaſe exiſted in the habit, had thrown a languor upon every exertion which, in the earlier ſtages of the complaint, might have been attempted for the relief of the patient. The objection to an operation was now, in ſome meaſure, done away by the diſappearance of the tumour in the left ham; but ſtill the great bulk of the aneuriſm, the abſolute impoſſibility of fixing a ligature low enough to give a chance of preventing the limb from becoming becoming mortified, and the difficulty of compreffing the artery fo as to prevent a fatal hemorrhage, were fufficient reafons for not attempting an uncertain operation.

The tumor was covered with a pledgit of white cerate. Opiates, gentle laxatives, and occafional venæfection contributed, for about fix weeks, to palliate the complaint; but, at the end of that time the inflammation became livid, and a fphacelus, which took place on the integuments, terminated his life without any hæmorrhage.

The ftate of the limb was examined the day after his death, in the prefence of feveral of the gentlemen above mentioned, and of Mr. Watfon, furgeon of the Weftminfter hofpital. The diffection exhibited the ufual aneurifmal appearances. The coats of the artery had given way, and burft at the fore part of the veffel; but the blood was prevented from iffuing externally by the thick coagulum which adhered to the inflamed and fphacelated integuments, and formed a pretty ftrong barrier againft the force of the blood.

The internal part of the aneurifmal fac was lined with ftrata of coagulable lymph adhering to the dilated veſſel, and in ſome parts theſe layers were three inches in thickneſs. We found alſo, by the diſſection, that the dilatation had taken place about an inch and a half below the arteria profunda, which veſſel was in a perfectly healthy ſtate. About two inches only, in length, of the femoral artery had been expanded, ſo as to form the tumour, extending as before deſcribed; the whole of the artery, both above and below this part, being quite free from diſeaſe. The blood ſeemed to have paſſed through the center of this maſs, as there was neither any diminution of the femoral artery below the aneuriſm, nor any enlargement of the arteria profunda; and the degree of œdema, or coldneſs, in the foot, was not greater than might be ſuppoſed to proceed from preſſure.

The aneuriſm of the left ham became now the ſubject of inveſtigation. Externally there was no mark left of the tumour; but, upon cutting down to the veſſel, we found the popliteal artery enlarged to the ſize of a ſmall hazle nut. On opening the artery, both above and below this tumour, and endeavouring to paſs a director and a probe, it was found to be quite impervious to the inſtruments, although

ſome

ſome force was uſed; and upon farther examination it was found plugged up by a ſubſtance of a hard and firm conſiſtence *.

### CASE II.

In the preceding caſe we have ſeen the ſpontaneous efforts of the conſtitution ſtruggling, in vain, againſt an improper mode of treatment, or perhaps againſt the irreſiſtible violence of the diſeaſe: in the preſent inſtance, I am happy in being able to relate a more ſucceſsful event. The diſeaſe, in this caſe, put on the ſame formidable appearance; but nature, by being left undiſturbed, produced a more favourable termination.

James Robſon, aged thirty-ſeven years, and of a fair complexion, applied to me on the 24th of September, 1785, for the cure of a ſwelling on his thigh. On examination, I found it clearly aneuriſmal, being attended with ſtrong pulſation, and an œdematous ſwelling of the foot and leg. It was now of about the ſize of a middle-ſized China orange, and obviouſly increaſing. The ſituation of it was ſo high up

* This portion of the popliteal artery is now in my poſſeſſion.

as to admit of no hope of preferving his life by removing the limb, or by tying up the artery. It was therefore only recommended to him to lie in bed, to keep his bowels open by gentle laxatives, and to live upon a very fpare diet.

He was feen by feveral gentlemen of the profeffion, amongft whom were Dr. Jackfon, Mr. Hawkins, Mr. Home, Mr. John Howard, Mr. Hunter, Mr. Pearfon, and Mr. Vaux, all of whom were convinced of the nature of the difeafe, and of the impracticability of affording the patient any affiftance by the operation ufual for aneurifms. With the concurrence, however, of thofe gentlemen, a compreffion was endeavoured to be made upon the artery in the groin; but the pain it occafioned in the limb, when the compreffion was ftrong enough to reftrain the pulfation of the tumour, foon obliged us to forego this experiment. The difeafe was now left wholly to itfelf, and for four months thofe fymptoms continued to prevail which ufually precede a fatal termination. His pulfe was hard and full, and the fwelling, daily increafing, extended from Pouparts ligament upwards, almoft to the ham downwards; the knee was bent without a pof-

ſibility of extenſion; the leg and foot were cold and œdematous; the pulſation was ſtrong in every part of the tumour; and the ſkin was tenſe and inflamed, appearing as if ready to give way in various places. He lay, in this manner, immovable for a long time, daily expecting the fatal moment when the integuments would be ruptured, and a fatal hæmorrhage enſue.

At the end of ſix months, during which time I had frequently called upon him, he began to think that the pulſation was not ſo ſtrong in the ſwelling, and that it had now ceaſed to increaſe; for, having anxiouſly watched its progreſs, he had, during the whole of his illneſs, regularly meaſured it every week. Circumſtances ſo favourable encouraged him to continue the plan of reſt and abſtinence which had been enjoined him, and it proved ſo ſucceſsful, that, in the month of March, the circumference of the tumour was much leſſened, and the pain had ceaſed; the tenſion was alſo diminiſhed; the inflammation of the ſkin had given way, and was now become ſcabrous, putting on a mottled look, and appearing in ſome parts brown, and in others of an orange colour. He had alſo now the power of extending ing ing his knee a little, and the coldneſs and ſwelling of the foot were going off. For two months afterwards the tumour continued to leſſen. His diet was gradually mended; a little animal food was occaſionally allowed him, and he began to gain ſtrength, and to ſit up in his bed; and as ſoon as he could be removed with ſafety, he was ſent into the country, where he ſoon recovered his ſtrength and the uſe of his limbs ſo much, that in three months he was able to walk ſeveral miles with a ſtick. It is now upwards of two years ſince he has been able to reſume his buſineſs, which is that of a ſtay maker. He frequently walks ten miles in a day without feeling any inconvenience, neither his leg nor his foot ſwelling. The thigh is two inches and a half in circumference larger than the other, and there is a hard incompreſſible tumour where the aneuriſm was, but which gives him no uneaſineſs.

REMARKS.

The hiſtories of uncommon caſes, particularly of ſucceſsful ones, are perhaps not the moſt uſeful communications in ſurgery. The feelings of human nature ſtrongly militate againſt chirurgical operations, and the report of one limb being preferved from amputation has frequently occafioned the lofs of many lives. It is, however, a fact too well authenticated, that, in the cafe of a femoral or popliteal aneurifm, neither the operation of tying the artery in the ufual mode, nor even that of amputating the limb, can be claffed among the moft fortunate efforts of furgery. We cannot be furprifed, therefore, to fee endeavours ufed to leffen the hazard of the operation, in fuch cafes, by an improved mode of performing it; or to render it unneceffary, by purfuing the indications of nature in the cure of aneurifm. That fuch means have been fuccefsfully purfued, is obvious from the cafes of aneurifm recorded by Morgagni * from Valfalva; but the manner in which the cures were performed being then unknown, they appear with an air of empiricifm, and we hefitate to believe what we are not able to explain. Many writers, however, fince that time fpeak of the poffibility of curing an aneurifm, and various remedies are propofed by them for this purpofe; but ftill they place their chief reliance on bleeding, laxatives, and a low regimen,

* De fedibus & caufis Morborum, Epift. iv. art. 9. Epift. xvii. art. 30.

men, and on keeping the patient quiet. This plan, I presume, they have recommended from observing that nature has sometimes effected a cure when such a mode has been adopted. Some cures of aneurism in the trunk, by medicine, are mentioned in books; but not being supported by any principles of anatomy, their authenticity must be very doubtful. It is certain, however, that an artery may remain dilated for many years before the fatal catastrophe takes place; and, if proper precautions are used, the coats of the vessel, though they have given way in some particular part, may, for a long time, sustain the impetus of the blood.

In Guattani's work * upon this subject we have a series of well-supported facts relative to aneurism of the extremities. Some cases are related wherein the cure could be ascribed only to the spontaneous efforts of nature; in others the complaint was removed by compresses and bandages applied above the diseased part. Upon the whole, I think it may be presumed, from the cases above described, and from others

* De Externis Aneurismatibus, Hist. 3, 4, 6, & 7.

which are related by authors, and in a very circumſtantial manner by Guattani,

1ſt, That nature is capable of effecting the cure of many aneuriſms ſolely by her own efforts.

2dly, That theſe efforts have been ſucceſsful even when counteracted by improper treatment, as in the caſe of the popliteal aneuriſm, of Cathay, above related; but that a quiet poſition of the limb, with an antiphlogiſtic regimen, contributes to the cure.

3dly, That the cure by nature is a permanent one.

4thly, That the inert maſs left behind is not likely to produce any miſchief.

5thly, That the unſucceſsful event of the operation, for the popliteal aneuriſm, does not, principally, depend on any particular hazard in conſequence of an obſtructed circulation in the ham, but upon other cauſes.

With regard to the manner in which the coaleſcence of the artery is formed, I ſhould conſider it as a conſequence of the accumulation of ſtrata of coagulable lymph with which the aneuriſmal ſac is uſually lined: theſe ſtrata ſeem to be depoſited, from time to time, till, at length, in ſome caſes, they completely fill up the bag:

whenever this happens, if the communicant branches above the tumour are large enough to carry on the circulation in the extremity, the patient may (as in the cafe of Robfon) recover; but if they are not, a mortification muft, of courfe, enfue.

*Golden Square,*

May 22, 1788.

